# Molecular detection of Mycobacterium tuberculosis in pulmonary and extrapulmonary samples in a hospital-based study

**DOI:** 10.4314/ahs.v20i4.14

**Published:** 2020-12

**Authors:** Kalal Iravathy Goud, Matam Kavitha, Adi Mahalakshmi, Ravi Vempati, Abdulaziz A Alodhayani, Arif A Mohammed, Imran Ali Khan

**Affiliations:** 1 Molecular Biology and Cytogenetics Department, Apollo Hospitals, Jubilee Hills Hyderabad-500096, Telangana, India; 2 Family & Community Medicine Department, College of Medicine, King Saud University, Riyadh, Saudi Arabia; 3 Center of Excellence in Biotechnology Research, King Saud University, PO Box-2455, Riyadh, Saudi Arabia; 4 Department of Clinical Laboratory Sciences, College of Applied Medical Sciences, King Saud University, P.O. Box-10219, Riyadh-11433, Saudi Arabia

**Keywords:** Tuberculosis (TB), EPTB, PTB, Mycobacterium tuberculosis (Mtb)

## Abstract

**Objective:**

Tuberculosis (TB), caused by Mycobacterium tuberculosis (Mtb), remains a deadly infectious disease. India contributes to one-third of the global TB burden. However, no studies have been carried out in the Telangana (Hyderabad) population using real-time polymerase chain reaction (RT-PCR). Therefore, the current study evaluated the role of RT-PCR as a rapid and non-invasive test to diagnose TB by testing for pulmonary tuberculosis (PTB) and extrapulmonary tuberculosis (EPTB).

**Materials and methods:**

This hospital-based study examined 1670 samples (900 EPTB; 770 PTB) comprising tissue (n = 537), peritoneal fluid (n = 420), sputum (n = 166), bronchial fluid (n = 126), cerebrospinal fluid (n = 145), ascetic fluid (n = 76), sputum pus (n = 78), urine (n = 79), and bronchoalveolar fluid (n = 43) samples. DNA from samples was separated using specific isolation kits and subjected to RT-PCR.

**Results:**

In this study, we enrolled 1670 subjects and categorized 54.4% as females and 45.6% as males. The collected samples were categorized as 48.5% of fluid samples, followed by tissue (32.2%), sputum (9.9%), urine (4.7%), and pus-swab (4.6%). RT-PCR analysis revealed that 4.7% patients were positive for Mtb. Our results revealed that 61% of the affected patients were male and 39% were female. Among the specimen types, tissue samples gave the highest proportion of positive results (36.3%).

**Conclusion:**

The results showed that RT-PCR should be implemented and given top priority in TB diagnosis to save time and facilitate a definitive diagnosis. Tissue samples are highly recommended to screen the Mtb through the technique RTPCR. Future studies should extend the technique to the global population and exome sequencing analysis should be performed to identify TB risk markers.

## Introduction

Tuberculosis (TB), caused by acid-fast aerobic bacteria comprising the Mycobacterium tuberculosis (Mtb) complex, is a contagious infection that generally affects the lungs. TB is a communicable infectious disease that is transmitted through cough aerosols, and is characterized pathologically by necrotizing granulomatous inflammation, usually in the lungs 1. In 2015, 10.4 million new active TB cases were registered according to the World Health Organization (WHO) and 1.8 million deaths were documented^2^. TB has remained one of the major causes of morbidity and mortality in low-to-middle income countries 3. The risk factors for TB are: patients infected with HIV, diabetes, alcoholism, leukemia and patients who receive immunosuppressive drugs^4^,^5^. TB is usually diagnosed through clinical findings of chest radiography and microbiological tests. Smear microscopy of either sputum or tissue, and microbial cultures are used to confirm TB 6. Ongoing treatment for drug-susceptible TB comprises a four-drug regimen of isoniazid, rifampin, pyrazinamide, and ethambutol or streptomycin, which are administered for 6 months. Currently, new pharmaceutical agents are urgently required to control TB because of the increasing incidence of drug-resistant TB, multidrug-resistant TB (MDR-TB), and extensively drug-resistant TB (XDR-TB), which pose a major public health problem worldwide 7. Pulmonary tuberculosis (PTB) and extrapulmonary tuberculosis (EPTB) are the two clinical manifestations of TB. The involvement of TB other than lungs is termed as EPTB. Any patient affeted with the combination of pulmonary and EPTB is defined as PTB 8. In cases of suspected EPTB, rapid and accurate laboratory diagnosis is important, because traditional techniques of detecting acid-fast bacilli have limitations. During the last decade, remarkable progress has been made in the diagnostics of PTB. However, diagnostic challenges in EPTB remain to be addressed. A more accurate test to diagnose various forms of EPTB, which can easily be incorporated into the routine TB control program, would contribute significantly towards improving EPTB case-detection 9. EPTB constitutes about 15–20% of TB cases 10. The common cause of tuberculosis between human and animals is a group of mycobacterial species that form the Mtb complex 11. TB is a chronic disease and has long-lasting effects on the human body, with complications that are less common but life threatening 12. Molecular techniques have proven very successful as diagnostic tools. However, these molecular typing techniques target particularly polymorphic genetic sequences, but interrogate less than 1% of the genome; therefore, they have an intrinsically restricted discriminatory power. This limitation could be overcome by the application of next-generation whole-genome sequencing (WGS) for genome-based epidemiology. Various platforms for WGS have been developed in the last decade 13. Therefore, the current study aimed to evaluate the role of real-time polymerase chain reaction (RT-PCR) to test PTB and EPTB specimens as a rapid and non-invasive test for the fast diagnosis of TB.

## Methods

### Subjects

This was a hospital-based study carried out in Hyderabad, the capital city of Telangana, India. In this study we selected 1670 cases, of whom 770 were PTB and 900 were EPTB. The patients were recruited after an ethical grant (AHJ-114/10-18) from the Apollo Research committee to the hospital. The samples included tissue (n = 537), peritoneal fluid (n = 420), sputum (n = 166), bronchial fluid (n = 126), cerebrospinal fluid (n = 145), ascetic fluid (n = 76), sputum pus (n = 78), urine (n = 79), and bronchoalveolar fluid (n = 43) samples. The patients were recruited from January 2012 to March 2017. All subjects gave their consent to participate in the study. Sample processing differed according to the specimen type. Non-sterile samples were subjected to standard N-acetyl cysteine sodium hydroxide decontamination. The average duration of symptoms was 113 days (range, 35–320 days). Symptoms like uncontrolled cough for a minimum of 2 weeks with or without chronic fever or weight loss were evaluated for TB. The inclusion criteria of this study were: (i) suspected active TB; (ii) an age range of 16–60 years of age; (iii) no previous history of anti-TB treatment; and (iv) a negative status for HIV. The exclusion criteria were: (i) noinalized diagnosis after examination and treatment; (ii) previous identification of TB; and (iii) previous treatment for TB 14. Based on the typical TB clinical symptoms, all the patients were screened by bacterial culture and imaging examination. The acid-fast bacilli (AFB) culture results of these cases were positive, but some of the patients showed the negative TB clinical features. The patients had clinical and radiological features suggestive of TB. The AFB staining procedure was performed using N-acetyl-l-cysteine-NaOH, as described in an earlier publication 15. The formalin-fixed paraffin-embedded (FFPE) human specimens were examined after Ziehl-Neelsen (ZN) staining, which was performed according to standard protocols 16. All the slides stained with AFB were reconfirmed microscopically by two independent pathologists who were unaware of each patient's clinical details, as per the National Accreditation Board for Testing and Calibration Laboratories (NABL) protocol.

### DNA extraction

DNA was isolated from all the different types of samples using a QIAamp DNA mini kit (QIAGEN, Germany) according to the manufacturer's recommendations. For the FFPE specimens, 3-µm thick sections were cut from each paraffin block; from the resected specimens, three sections were taken; and from the biopsy specimen, five or more sections were taken. For the FFPE specimens, DNA was extracted using a QIAamp FFPE tissue kit (QIAGEN, Germany) and subsequently analyzed by PCR. The quantification of Mtb DNA was performed using an RG PCR kit, based on the manufacturer's instructions (QIAGEN, Germany). This kit detects specific variants appearing in the Mtb complex, including M. tuberculosis, M. africanum, M. bovis, M. bovis BCG, M. microti, and M. pinnipedii, using a single primer. Using Rotor-Gene Q with 5-plex HRM (Qiagen), RT-PCR was performed in a total volume of 25 µl, comprising 15 µl of master-mix, 7 µl of double distilled water, and 3 µl of template DNA. The PCR conditions were as follows: 2 min at 95 °C; followed by 45 cycles of 15 sec at 95 °C, 30 sec at 64 °C, and 20 sec at 72 °C. Standard curves from four quantification standards were used to calculate the amount of Mtb DNA (copies/µl).

## Results

In this hospital-based study, 1670 patients were recruited, of whom 45.6% were male and 54.4% were female. The mean age of the 1670 subjects was 41.36 ± 17.76 years; amongst, males were 47.87±18.39 years old and females were 36.28 ± 15.46 years' old (Table 1). The total samples were categorized as 53.2% were fluid samples, followed by tissue (32.2%), sputum (9.9%), and pus-swab (4.6%) (Table 1). The total number of fluids used in this study are 48.5% (n=810) and amongst them fluids are sub-categorized as follows; peripheral fluid (n=420; 25.2%), bronchial fluid (n=126; 7.5%), cerebrospinal fluid (n=145; 8.7%), ascetic fluid (n=76; 4.6%), bronchoalveolar fluid (n=43; 2.6%) and urine fluid (n=79; 4.7%). In this study, only 4.6% of the samples were found to be Mtb-positive by RT-PCR. The maximum number of positive samples were found among tissue samples (36.3%) followed by peritoneal fluid samples (27.3%), sputum (10.4%), cerebrospinal fluid (7.7%), bronchial fluid (6.5%), ascetic fluid (3.8%), pus-swab (3.8%), urine (2.5%), and bronchoalveolar fluid (1.2%) samples. The complete details of are shown in Table 2. We then classified the positive results based on the patient's gender in Table 3. Of the positive results obtained by RT-PCR, 61% were obtained for samples from male patients and 39% for samples from female patients. Among the tissue samples, more Mtb positive samples were obtained from female patients (64.3%) compared with those from male patients (35.7%). From the Mtb positive pleural fluid samples, 71.4% were obtained from male patients and 28.6% from female patients. All the positive samples from sputum, urine, and bronchoalveolar fluid were obtained from male patients. From the Mtb positive bronchial fluid samples, 60% were obtained from male patients and 40% from female patients. From the Mtb positive cerebrospinal fluid, ascetic fluid, and pus-swab samples, 66.7% were obtained from male patients and 33.3% from female patients. For the male patients, the highest proportion of positive RT-PCR results was obtained for peritoneal fluid (31.9%) and tissue (21.3%), samples whereas for the female patients, the highest proportions of positive results were obtained for tissue (60%) and peritoneal fluid (20%) samples.

**Table 1:** List of types of samples involved in this study

Sample Type	Total samples (n = 1670)	Positively Subjects (n = 77)	Negatively Subjects (n = 1593)
Tissue	537 (32.2%)	28 (36.4%)	509 (32%)
Fluids	810 (48.5%)	36 (46.7%)	774 (48.6%)
Sputum	166 (9.9%)	8 (10.4%)	158 (9.9%)
Pus Swab	78 (4.6%)	3 (3.9%)	75 (4.7%)
Urine	79 (4.7%)	2 (2.6%)	77 (4.8%)

**Table 2:** Detailed list of samples involved in this study

Type of Sample	Total no. of samples (n=1670)	Samples detected as positive (n = 77)
Tissue	537 (32.1%)	28 (36.3%)
Pleural fluid	420 (25.2%)	21 (27.3%)
Sputum	166 (9.9%)	8 (10.4%)
Bronchial fluid	126 (7.5%)	5 (6.5%)
Cerebrospinal fluid	145 (8.7%)	6 (7.7%)
Ascetic fluid	76 (4.6%)	3 (3.8%)
Pus Swab	78 (4.7%)	3 (3.8%)
Urine (fluid)	79 (4.7%)	2 (2.5%)
Bronchoalveolar fluid	43 (2.6%)	1 (1.2%)

**Table 3:** Mtb positive samples analyzed by gender

Type of Sample	Positive samples (n = 77)	Males (n = 47)	Females (n = 30)
Tissue	28 (36.3%)	10 (21.3%)	18 (60%)
Pleural fluid	21 (27.3%)	15 (31.9%)	6 (20%)
Sputum	8 (10.4%)	8 (17%)	0 (0%)
Bronchial fluid	5 (6.5%)	3 (6.3%)	2 (6.7%)
Cerebrospinal fluid	6 (7.7%)	4 (8.5%)	2 (6.7%)
Ascetic fluid	3 (3.8%)	2 (4.3%)	1 (3.3%)
Pus Swab	3 (3.8%)	2 (4.3%)	1 (3.3%)
Urine	2 (2.5%)	2 (4.3%)	0 (0%)
Bronchoalveolar fluid	1 (1.2%)	1 (2.1%)	0 (0%)

## Discussion

In this hospital-based study, among 1670 patients enrolled based on the symptoms of PTB and EPTB, 54.5% were female and 45.6% were male. Among the RT-PCR positive cases, 61% were male and 39% were female. In the present study, we detected TB in 4.6% of the patients using RT-PCR analysis. Tissue samples showed the highest rate of RT-PCR positivity, followed by various fluid samples. TB is an infectious disease; frequently affects the lungs, though it has ability to affect any organ in the body. Although, TB can be fatal and in numerous cases it was prevented and treated. The disease TB progresses which bacteria spread through droplets in the air. The effect of TB in other organs than lungs such as abdomen, bones, genitourinary tract, joints, lymph nodes, meninges, and pleura are defined as EPTB and a person combined with pulmonary and EPTB is confirmed as a case of PTB8. In this study, 900 subjects had PTB and 770 had EPTB. TB, an airborne disease, is one of the major infectious diseases worldwide and its prevalence varies in the different regions and ethnicities. According to WHO, 3.9% and 21% formerly untreated and treated cases, respectively, of TB were detected in 2015 and it has the Multi-drug resistant and Rifampicin-resistant TB 17.

Babafemi et al^18^ has performed a meta-analysis studies with Mtb and RT-PCR. These meta-analysis reports were carried out with 31 different global studies and confirmed as RT-PCR assay can be used for screening the Mtb samples to diagnose the accurate results in couple of hours. The RT-PCR assay has confirmed high degree of sensitivity and for PTB and EPTB. Wang et al^19^ also implemented the similar study in Taiwanese population with RT-PCR in PTB and EPTB samples and confirmed as RT-PCR should be performed for the faster results. Our study is also in agreement with their studies. However, Raveendran et al^20^ performed the multiplex RT-PCR studies in only EPTB samples and he has concluded as multiplex RT-PCR can play an important role for accurate diagnosis of EPTB.

Worldwide, India ranks first in the prevalence of TB, followed by Indonesia, China, Philippines, Pakistan, Nigeria, and South Africa. However, in the Asian continent, 45% of the new cases of TB occur, followed by 25% in Africa. Recently, TB has become a leading disease, with a death rate of 40% in patients that are HIV positive 21. In India, TB was confirmed in 2.8 million patients, which accounts for 10.4% of the new cases in the global population. Worldwide, the death rate from TB was documented as 29% (1.8 million deaths). The death rate in India according to age-standardized TB was high from 2001–2007, but decreased from 2008 to 2013. Indian studies have documented associations between TB and (i) malutrition and tobacco smoking, (ii) lack of investment in health care, (iii) non-implementation of quality-health systems, and (iv) in the private sector, poor care of patients with TB 22. By contrast, in countries such as Saudi Arabia, the prevalence of EPTB was 28.4%, which is very low compared with that in India. The prevalence of TB in the Saudi national people was just 9% and in non-Saudi people in Saudi Arabia was 17.2%, mainly because of the Hajj and Umrah pilgrimages. However, in males, the prevalence was found to be high (57.5%) 23,24. The major differences between India and Saudi Arabia are the sizes of their populations: India has 1.324 billion people and Saudi Arabia has 32.28 million; and Saudi Arabia spends more money on hospitals in both the public and private sectors to control TB.

The diagnosis of TB depends mainly on chest X-ray, bacterial cultures, tuberculin tests, ZN microscopy, nucleic acid amplification, or RT-PCR tests. However, serological tests have been banned in India because of the high prevalence of antibodies in the blood. Mtb that survives in the human body and does not infect other persons, and shows no signs and symptoms, is defined as latent TB. Latent TB is not contagious and is not treated in India because 40% of the population have the infection but not the disease. Only active TB is considered as a clinical condition and this leads to ineffective or incurred diagnosis. Chronic bacterial infection caused by Mtb is defined as active TB. However, based on blood tests, the antibody response levels cannot be differentiated between active and latent TB.

Advanced technologies should be applied for the prompt diagnosis of TB. Previous studies have documented the importance of skilled trained technicians, tools, and the time available to perform tests for TB. The duration of cultural tests is very sensitive for obtaining accurate results and often demands additional cultures. However, sputum tests produce results in 30 min in 10–75% of TB cases when performed by expert microscopists. Although India has skilled staff with expertise in handling the diagnostic tools, the diagnosis and treatment of TB is not often quick enough; thus, the disease spreads quickly, especially in crowded areas^25^–^27^. For TB, molecular diagnosis was reported early in the 1990's by Hance et al 28, who described a specific and sensitive PCR technique that is capable of detecting ∼100 ng of purified DNA of Mtb. Accurate analysis by PCR was provided by Rondini et al 29 through screening of Mtb samples using RT-PCR. Earlier studies have already documented that PCR for Mtb is a powerfl and useful tool in the diagnosis of infectious TB 16. However, recent studies have shown that RT-PCR is a more advanced method to screen for TB 15,30–35.

WGS is the process of reading the complete DNA sequence of an organism (i.e., screening of genetic material). In Mtb, the WGS means decoding the exact nucleotide sequence from bacterial genome^36^. Next-generation whole genome sequencing (NG-WGS) of TB has been implemented earlier but its application in the clinical practice is still very limited. The WHO has been recognized NG-WGS has great potentiality for diagnosing drug-resistant TB in diverse clinical settings. NGWGS allows the researchers to generate millions of genome-sequences in a short-time. This technique in the diagnosis of Tb identifies the accurate type of Mtb affecting the patient which involves the drug resistance profile which allows clinicians to recognize the suitable treatment regimen to combat the disease^37^.

The strength of the current study was that it analyzed a relatively large number (1670) of cases with symptoms of TB, and diagnosis was carried out using RT-PCR. A limitation of our study was that we did not take into account the microbiological results, TB culture result, and patients' clinical details.

## Conclusion

The study results showed RT-PCR was used to screen the PTB and EPTB patients and our study has showed 4.7% were positive for Mtb. Our study also confirms RT-PCR has an ability and latent to increase the rapid diagnosis of PTB and EPTB. Future studies with RTPCR should be implemented and given top priority for a faster initial diagnosis of TB so that patients requiring further diagnostic tests may be identified. Tissue samples are highly recommended to screen the Mtb through the technique RT-PCR. We recommend RT-PCR, molecular techniques should be implemented in the global population, and future studies should perform second generation sequencing analysis to identify risk markers for TB.
